# Risk of Esophageal and Gastric Cancer by Histologic Subtype in Steatotic Liver Disease: A UK Biobank Study

**DOI:** 10.3390/cancers17213416

**Published:** 2025-10-24

**Authors:** Donghoon Kang, Ji Won Han, Kenneth R. Muir, Artitaya Lophatananon, Jongin Lee

**Affiliations:** 1 Department of Internal Medicine, Seoul St. Mary’s Hospital, College of Medicine, The Catholic University of Korea, Seoul 06591, Republic of Korea; etiria@catholic.ac.kr (D.K.); tmznjf@catholic.ac.kr (J.W.H.); 2 The Catholic University Liver Research Center, Department of Biomedicine & Health Sciences, College of Medicine, The Catholic University of Korea, Seoul 06591, Republic of Korea; 3 Division of Population Health, Health Services Research and Primary Care, School of Health Sciences, Faculty of Biology, Medicine and Health, The University of Manchester, Manchester M13 9PL, UK; kenneth.muir@manchester.ac.uk (K.R.M.); artitaya.lophatananon@manchester.ac.uk (A.L.); 4 Department of Occupational and Environmental Medicine, Seoul St. Mary’s Hospital, College of Medicine, The Catholic University of Korea, Seoul 06591, Republic of Korea

**Keywords:** steatotic liver disease, esophageal cancer, gastric cancer, alcohol consumption, UK biobank

## Abstract

Aim: The recently updated steatotic liver disease (SLD) nomenclature provides a refined classification accounting for both metabolic dysfunction and alcohol exposure. However, the relationship between SLD subtypes and upper gastrointestinal (UGI) cancer risk remains unclear. Methods: We analyzed 456,367 UK Biobank participants, categorizing them into non-SLD, metabolic dysfunction-associated steatotic liver disease with no alcohol (MASLD1), MASLD with minimal alcohol use (MASLD2), metabolic dysfunction-associated alcohol-related liver disease (MetALD), and alcohol-associated liver disease (ALD) groups. Cox proportional hazards models estimated cancer risks over a median 13-year follow-up, with histologic subtype-specific analyses. Results: Compared to non-SLD, esophageal cancer risk was significantly elevated in all SLD groups with any alcohol consumption (MASLD2, MetALD, and ALD), but not in purely metabolic SLD without alcohol (MASLD1). This association was driven by adenocarcinoma subtype, with hazard ratios ranging from 1.67 to 1.80 in alcohol-exposed SLD groups. For gastric cancer, elevated risk was observed primarily in ALD and MetALD groups, affecting intestinal-type cancers. Squamous cell esophageal cancer and non-intestinal gastric cancer showed no significant associations. Conclusions: Upper GI cancer risk in SLD patients is significantly modified by alcohol consumption, with combined metabolic dysfunction and alcohol exposure conferring the highest risks for esophageal adenocarcinoma and intestinal-type gastric cancer. Clinically, these findings suggest that SLD patients with any level of alcohol consumption require heightened cancer surveillance. Even minimal alcohol intake substantially increases cancer risk in metabolically compromised individuals, supporting alcohol reduction as a key preventive strategy.

## Simple Summary

Steatotic liver disease (SLD), formerly known as fatty liver disease, affects over 30% of the global population and has been linked to cancers outside of the liver. The recently updated classification system distinguishes between SLD subtypes based on alcohol consumption patterns. While previous studies have reported a relationship between these refined SLD classifications and extrahepatic cancer risk, the specific histologic cancer subtypes were unclear. This study’s findings revealed that alcohol consumption significantly modifies cancer risk in SLD patients. Those with combined metabolic dysfunction and alcohol exposure showed the highest risk of esophageal adenocarcinoma and intestinal-type gastric cancer. Other subtypes, such as squamous esophageal cancer and non-intestinal gastric cancer, did not show a clear association with SLD. Furthermore, purely metabolic SLD without alcohol consumption was associated with minimal cancer risk elevation, whereas even minimal alcohol consumption substantially increased risk in SLD patients.

## 1. Introduction

Recently, non-alcoholic fatty liver disease (NAFLD) was officially renamed metabolic dysfunction-associated steatotic liver disease (MASLD) to highlight the disease’s underlying metabolic risk factors and reduce the stigma associated with the term ‘fatty’. Under this new framework, steatotic liver disease (SLD) is the overarching term, with subcategories defined in part by alcohol consumption thresholds [1]. MASLD specifically denotes SLD with low or no alcohol intake, while patients with moderate alcohol consumption are now classified as having metabolic dysfunction-associated alcohol-related liver disease (MetALD). Cases of steatosis driven primarily by heavy alcohol consumption continue to be termed alcoholic liver disease (ALD). This refined classification is clinically meaningful because it acknowledges the common overlap of metabolic and alcohol-related causes, thereby enabling more nuanced patient stratification and research [2].

Recent studies indicate that hepatic steatosis itself, together with metabolic disturbances such as insulin resistance and chronic inflammation, can promote carcinogenesis by driving oxidative stress, reprogramming lipid metabolism, and creating a pro-inflammatory microenvironment [3]. Notably, epidemiological studies have linked MASLD to upper gastrointestinal (GI) cancers. A previous study demonstrated that MASLD is associated with significantly higher risks of extrahepatic malignancies including upper GI cancers compared to individuals without MASLD [4], as well as multiple cancers of non-gastrointestinal origin [5]. In a recent Korean cohort study, patients with MASLD were found to have a higher incidence of GI cancers, including esophageal and gastric malignancies, than the control group [6]. However, some studies have found no significant increase in gastric cancer risk with MASLD patients [7], pointing to possible heterogeneity in terms of the MASLD population and cancer subtypes.

Apparently, upper GI cancers are heterogeneous, with distinct histologic subtypes that differ in etiology and risk factors. Esophageal adenocarcinoma is driven predominantly by obesity-related reflux, whereas squamous cell carcinoma is linked to chronic alcohol exposure [8]. Gastric cancers are also split into intestinal and diffuse types under Lauren’s classification, with the former associated with metabolic and environmental factors, including alcohol consumption, and the latter more linked to genetic predisposition and H. pylori infection [9]. Furthermore, upper GI cancers are strongly influenced by alcohol intake because upper GI tract is the first anatomical site to directly encounter ingested alcohol, making it particularly susceptible to alcohol-induced carcinogenesis [10]. Esophageal cancer has ethanol as a major etiological factor [11], and even moderate drinking has been associated with an increase in gastric cancer risk [12]. Therefore, when evaluating the relationship between SLD and cancers of the esophagus or stomach, it is critical to account for stratified analyses by alcohol exposure and cancer subtypes when assessing upper GI cancer risk in this context.

In the present study, we investigated the risk of upper GI cancers in patients with SLD. We analyzed a large population-based cohort, comparing the incidence of upper GI cancer and its subtypes among individuals categorized as MASLD, MetALD, or ALD, with stratification by alcohol consumption. This approach allows us to disentangle the contributions of metabolic dysfunction versus alcohol exposure to upper GI cancer development. By leveraging the updated SLD nomenclature, our study aims to clarify the independent upper cancer risks associated with SLD and illustrate the utility of the new sub-classification in clinical risk stratification.

## 2. Materials and Methods

### 2.1. Study Design

This was a retrospective population-based cohort study utilizing data from the UK Biobank, a large-scale prospective cohort established to investigate the determinants of diseases in middle-aged and older adults [13]. The UK Biobank represents a closed cohort design with fixed enrollment between 2006 and 2010, followed by passive surveillance through linkage to national health registries. Participants were recruited from the general population across the United Kingdom and underwent comprehensive baseline assessments at designated assessment centers. Following initial enrollment, participants’ health outcomes were monitored through regular linkage to national cancer registries, death registries, and hospital episode statistics, rather than through active follow-up visits. This passive surveillance approach enabled long-term tracking of cancer incidence and mortality outcomes with minimal loss to follow-up. The cohort entry date was defined as each participant’s first assessment center visit date, and follow-up continued until the occurrence of cancer diagnosis, death, or the end of the study period, whichever occurred first.

### 2.2. Study Participants

**Figure 1:**
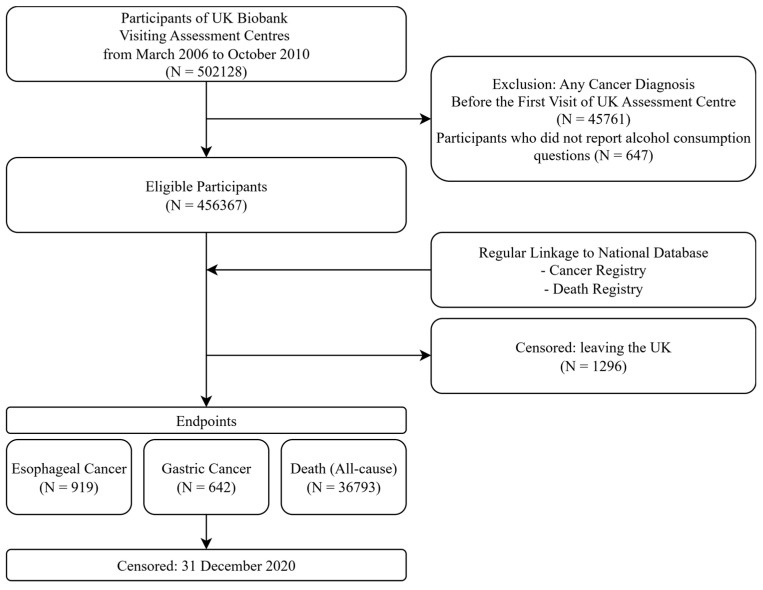
Study Population Selection and Follow-up Scheme for This Study in the UK Biobank Cohort.

From the total UK Biobank cohort of 502,128 participants, we applied specific inclusion and exclusion criteria, as outlined in Figure 1. All participants in the UK Biobank were eligible for inclusion, with the actual age distribution ranging from 37 to 73 years (median 58 years). Inclusion criteria were: (1) availability of complete data for fatty liver index (FLI) calculation, including body mass index, waist circumference, triglycerides, and gamma-glutamyl transferase and (2) complete alcohol consumption frequency data. Exclusion criteria included (1) participants with a prior cancer diagnosis before their assessment center visit (n = 45,761) and (2) participants who selected “Prefer not to answer” for alcohol consumption questions (n = 647), as these responses could not be classified under the SLD framework. After applying these criteria, 456,367 participants were eligible for analysis.

At the baseline assessment center visit, participants underwent comprehensive evaluations, including demographic questionnaires, anthropometric measurements, blood sampling for laboratory tests, and detailed lifestyle assessments, including alcohol consumption patterns. All baseline characteristics and exposure variables used in this study were collected during this single assessment center visit.

### 2.3. Definition of Cancer Diagnosis

The UK Biobank participants’ cancer diagnosis data has been regularly linked to the cancer registry. This work uses data provided by patients and collected by the NHS as part of their care and support. The censoring dates are 31 December 2020 (data from NHS England) and 30 November 2021 (data from National Records of Scotland or NHS Central Register). Therefore, the study participants’ first assessment centre visit date was set as the cohort entry date, and the date of the first cancer diagnosis was set as the endpoint. Since the data was also linked to the death registry, if the date of death was confirmed, the date of death was set as the endpoint where no event occurred. If neither cancer diagnosis nor death was confirmed, the final endpoint (censoring) was the censoring dates of each cancer registry resource. In patients who experienced more than one cancer, diagnoses were recorded sequentially according to the order of diagnosis confirmed in the cancer registry, with each participant assigned an instance number. Only the first cancer diagnosis in a participant’s lifetime was set as the endpoint, and secondary cancers occurring after the initial cancer diagnosis were excluded from the study. The minimum and maximum follow-up periods were 0 months and 16.6 years, respectively, with a median of 12.96 years and an average of 12.08 years. Cancer diagnoses were classified using ICD-10 codes (C15 for esophageal and C16 for gastric cancer) in the cancer registry. A detailed analysis by histological type was conducted using the ICD-O-3 morphology codes, categorizing esophageal cancers into squamous cell carcinoma (8070, 8071, 8072, and 8076), adenocarcinoma (8140, 8144, 8145, 8210, 8211, 8246, 8260, 8323, 8480, 8481, 8490, 8560, and 8574), and others, and gastric cancers into intestinal (8140, 8142, 8144, 8210, 8260, 8263, 8480, and 8481) and non-intestinal types.

### 2.4. Definition of Steatotic Liver Diseases

Steatotic liver diseases were defined using the fatty liver index (FLI). The FLI was calculated according to the definition by Bedogni et al. [14]. The UK Biobank includes a question asking how frequently participants consume alcohol. Responses were categorised as follows: “Never”, “Special occasions only”, “One to three times a month”, “Once or twice a week”, “Three or four times a week”, and “Daily or almost daily”. A total of 647 participants selected “Prefer not to answer”, and these responses were excluded from the analysis as they could not be included in the disease definition. The presence of SLD was determined based on an FLI of 60 or higher, along with body mass index (BMI), waist circumference, fasting blood glucose, hypertension, triglycerides, and high-density lipoprotein (HDL) cholesterol. Since SLD is clinically more significant in non-drinkers, cases that meet the above definition of SLD and have alcohol consumption of “Never” are classified as “MASLD1 (MASLD with no alcohol use)”; “Special occasions only”, “One to three times a month”, and “Once or twice a week” are classified as “MASLD2 (MASLD with minimal alcohol use)”; and ‘Three or four times a week’ and ‘Daily or almost daily’ are classified as ALD.

### 2.5. Statistical Analysis

According to the five classifications of SLD, we described the age, sex, smoking status, alcohol consumption frequency, presence or absence of hypertension, diabetes, and dyslipidemia, and biological markers (BMI, waist circumference, gamma-glutamyl transferase (GGT), fasting blood glucose, triglycerides, and HDL cholesterol) for each category. Differences between groups were tested using the chi-square test.

The occurrence of cancers by classification was described, and differences were tested using the chi-square test. The Cox proportional model was applied to estimate the hazard ratios (HRs) for each cancer type, treating death from other causes as a competing risk. Fine and Gray’s competing risk models were also applied to consider the competing risks, including death and diagnoses of other kind of cancers. Each survival analysis was conducted separately for univariate and multivariate analyses. In the multivariate analysis, all variables presented in the initial demographic characteristics were included. To identify a minimally sufficient set of confounders for multivariable adjustment, we constructed a directed acyclic graph based on prior knowledge and biologic plausibility (Figure S1). The DAG suggested that age, sex, smoking, alcohol consumption, and metabolic diseases act as common causes of both SLD and UGI cancers and were therefore included in the adjusted models. Multicollinearity among covariates was assessed using variance inflation factor (VIF) analysis, with GVIF^(1/(2 × Df)) values > 2.0 considered indicative of concerning multicollinearity. Because age is an important risk factor for cancer development, age-stratified analyses were performed using 60 years as the cut-off point to examine differential associations across age groups. Statistical significance was assessed based on a *p*-value < 0.05 or a 95% confidence interval (CI). All statistical analyses were performed using R version 4.4.2.

## 3. Results

### 3.1. Demographic and Clinical Characteristics

As summarized in Table 1, significant differences were observed in demographic and clinical characteristics among the study groups (non-SLD, MASLD1, MASLD2, MetALD, and ALD) (all *p* < 0.001). The non-SLD group included 293,032 participants, while MASLD1 had 13,996, MASLD2 had 79,472, MetALD had 35,905, and ALD included 33,548 individuals. The mean age was highest in the ALD group (58.10 ± 7.39 years) and lowest in MASLD2 (56.51 ± 8.01 years). Males predominated in the ALD (80.1%) and MetALD (75.3%) groups, whereas females predominated in non-SLD (63.4%), MASLD1 (53.4%), and MASLD2 (56.7%). Weekly alcohol consumption clearly defined subgroups, with MASLD1 reporting no alcohol intake, MASLD2 minimal alcohol intake (<2 times/week), MetALD moderate use (3–4 times/week), and ALD almost daily consumption, suggesting appropriate definition and distribution of study subgroups. Never-smokers comprised the majority in non-SLD (58.8%), MASLD1 (60.2%), MASLD2 (53.5%), and MetALD (46.1%), while current smokers represented 50.9% of the ALD group, with previous smokers at 35.0%. The prevalence of hypertension, diabetes, and dyslipidemia was highest in MASLD groups and lowest in the non-SLD group. BMI and waist circumference were notably higher in MASLD groups, whereas GGT levels peaked in ALD (72.85 ± 79.11 IU/L) and MetALD (57.26 ± 54.44 IU/L), were intermediate in MASLD groups, and were lowest in the non-SLD group (26.21 ± 20.62 IU/L).

### 3.2. Incidence of Upper GI Cancers During Follow-Up According to SLD Subgroups

As summarized in Table 2, over a median follow-up of 13 years, significant differences in incident UGI cancers were observed across the study groups (non-SLD, MASLD1, MASLD2, MetALD, and ALD). The non-SLD group exhibited the lowest incidence of esophageal cancer (12.08 per 100,000 person-years), with higher proportions observed in MASLD1 (23.73), MASLD2 (23.40), MetALD (24.91), and ALD (32.60). Within esophageal cancer subtypes, adenocarcinoma was more frequent than squamous cell carcinoma across all groups, with incidence rates of 7.12 (non-SLD), 16.43 (MASLD1), 20.22 (MASLD2), 19.51 (MetALD), and 23.36 (ALD) per 100,000 person-years, whereas squamous cell carcinoma occurred less frequently at 4.51, 5.48, 2.12, 4.00, and 7.70 per 100,000 person-years, respectively.

For gastric cancer, incidence was lowest in the non-SLD group (9.27 per 100,000 person-years) and highest in MASLD1 (22.51), with intermediate values in MASLD2 (15.03), MetALD (14.57), and ALD (19.00). Among gastric cancer subtypes, the intestinal type predominated across all groups, with incidence rates of 5.75 (non-SLD), 16.43 (MASLD1), 11.22 (MASLD2), 10.58 (MetALD), and 14.63 (ALD) per 100,000 person-years. In contrast, the non-intestinal type showed low incidence across groups, without a clear association with SLD status: 3.52 (non-SLD), 6.08 (MASLD1), 3.81 (MASLD2), 4.00 (MetALD), and 4.36 (ALD). These findings indicate that the elevated risk of UGI cancers in SLD populations might be driven by esophageal adenocarcinoma and intestinal-type gastric cancer, while the incidence of squamous cell carcinoma and diffuse-type gastric cancer remains relatively unchanged across groups.

### 3.3. Cumulative Incidence and Subtype-Specific Risk of Upper GI Cancers by SLD Category

**Figure 2:**
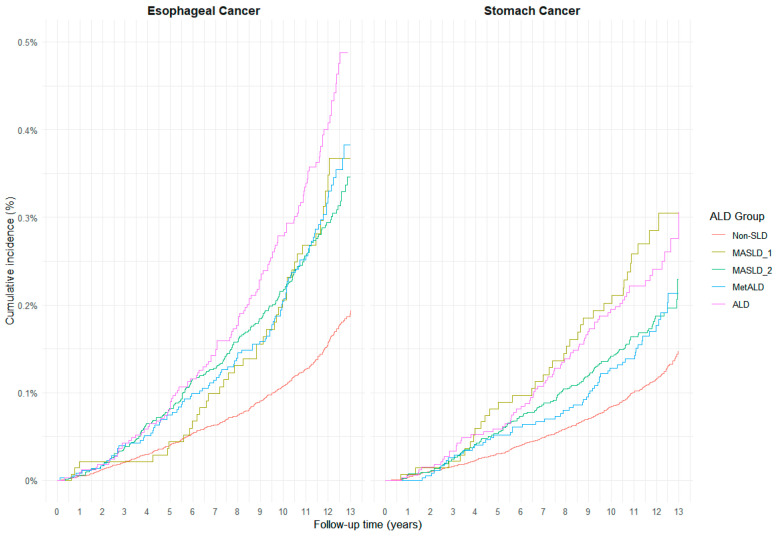
Cumulative Incidence of Esophageal and Gastric Cancers by MASLD Subtype Over 13 Years of Follow-up (Crude, Age-unadjusted).

As shown in Figure 2, cumulative incidence patterns for esophageal and gastric cancers differed by SLD category. For esophageal cancer, the ALD group exhibited a distinctly higher cumulative incidence compared to all other groups throughout follow-up. The MASLD1, MASLD2, and MetALD groups showed relatively similar trajectories, with modest elevations above the non-SLD group, which consistently had the lowest incidence. In contrast, for gastric cancer, the cumulative incidence was highest in the ALD and MASLD1 groups, which demonstrated nearly overlapping curves, whereas MASLD2 and MetALD showed intermediate incidence levels. These patterns suggest differential carcinogenic contributions of metabolic dysfunction and alcohol exposure depending on cancer type.

Further analyses highlighted associations between SLD subtypes and histological subtype-specific risks (Table 3 and Figure S2). For esophageal cancer, adjusted HRs analyzed by the Cox proportional hazards model showed a significantly increased risk in MASLD2 (HR 1.60, 95% CI: 1.30–1.96), MetALD (1.41, 1.07–1.86), and ALD (1.49, 1.14–1.93), whereas MASLD1 showed no significant association (1.00, 0.66–1.53). This elevated risk was primarily driven by the adenocarcinoma subtype, which demonstrated consistent and statistically significant associations in MASLD2 (1.77, 1.40–2.24), MetALD (1.80, 1.27–2.55), and ALD (1.67, 1.20–2.32), while MASLD1 (1.52, 0.86–2.69) showed a non-significant increase. In contrast, squamous cell carcinoma was not significantly associated with SLD in any group.

For gastric cancer, a statistically significant increase in overall risk was observed only in the ALD group (HR 1.55, 95% CI: 1.10–2.19). When stratified by histologic subtype, the intestinal type demonstrated significant associations in ALD (1.77, 1.17–2.68) and MetALD (1.74, 1.10–2.73), while MASLD2 (1.21, 0.92–1.60) and MASLD1 (1.67, 0.94–2.97) showed non-significant trends. In contrast, non-intestinal gastric cancer was not significantly associated with any SLD group. Although absolute estimates varied slightly, the direction and significance of associations were largely consistent between Cox proportional hazards models and Fine–Gray competing risk models, particularly for esophageal adenocarcinoma and intestinal-type gastric cancer (Table S1). These findings underscore the histologic specificity of cancer risk associated with SLD, particularly implicating esophageal adenocarcinoma and intestinal-type gastric cancer as the dominant subtypes affected by alcohol exposure. The absolute risk difference for esophageal adenocarcinoma and gastric cancer of intestinal type is presented in Table S2. Multicollinearity assessment revealed no concerning associations among variables (all GVIF^(1/(2 × Df)) < 1.5), supporting the stability of our multivariate model estimates (Table S3).

Age-stratified analyses revealed stronger associations between SLD subtypes and upper GI cancer risk in participants younger than 60 years compared to older participants (Table 4). In the younger group, esophageal cancer risks were significantly elevated across all SLD subtypes, with hazard ratios ranging from 1.51 (MASLD2) to 2.19 (MetALD). The association was particularly pronounced for esophageal adenocarcinoma, with significant increases in SLD groups: MASLD2 (1.49, 1.01–2.18), MetALD (3.11, 1.54–6.25), and ALD (1.96, 1.09–3.51). Similarly, intestinal gastric cancer showed significant associations across most SLD groups in younger participants.

In participants aged 60 years or older, associations were more modest but remained significant for esophageal cancer, primarily driven by adenocarcinoma subtype. For intestinal gastric cancer in older participants, significant associations were observed in MetALD group. Non-intestinal gastric cancer showed no significant associations in either age group.

## 4. Discussion

This large UK Biobank cohort study is, to our knowledge, the first to stratify esophageal and gastric cancer risk by both alcohol intake and SLD subtype using the new MASLD framework and histologic subtypes. A key strength of our analysis is the extensive sample size, as well as detailed phenotyping, which allowed refined classification of SLD [15]. By leveraging the updated definitions and examining cancer risk by histologic subtype, we provide novel insights into how metabolic dysfunction and alcohol synergistically influence GI carcinogenesis. MASLD (formerly NAFLD) is the most common chronic liver disease globally (>30% of the population) [16], yet its implications for extrahepatic cancers remain under-characterized. The present study’s design and scale address this gap, highlighting the population-level importance of our findings.

MASLD is emerging as a risk factor for extrahepatic malignancies, including UGI cancers, and population-based studies have shown that individuals with MASLD have higher incidences of esophageal and gastric cancers compared to metabolically healthy controls [17]. However, the new SLD framework highlights that alcohol exposure modifies this risk [18]. Under the updated nomenclature, refined classification acknowledges the common overlap of metabolic and alcohol etiologies and allows more nuanced risk stratification [19]. Indeed, patients with combined metabolic dysfunction and higher alcohol intake exhibit the greatest overall cancer risks. For example, those with metabolic dysfunction-associated fatty liver disease (MAFLD) with alcohol intake had a dramatically elevated hazard for esophageal cancer (~2.1-fold) relative to non-MAFLD, far higher than the slight risk increase seen in pure metabolic MAFLD [19]. These data suggest that while MASLD itself might confer some excess UGI cancer risk, the addition of alcohol exposure markedly amplifies the risk, underscoring the need to account for alcohol in evaluating cancer risk among SLD patients.

Esophageal and gastric cancers are heterogeneous in pathogenesis [20,21], so it is crucial to consider cancer subtypes when assessing the MASLD–cancer link. In our analysis, MASLD patients who consumed mild, moderate or heavy alcohol showed significantly higher incidence of esophageal adenocarcinoma, whereas those with purely metabolic MASLD without alcohol did not have a significant risk increase. A similar pattern emerged for intestinal-type gastric cancer—the risk was highest in ALD and MetALD groups, with pure MASLD showing little or no elevation in gastric cancer. Previous studies suggested that esophageal adenocarcinoma is driven predominantly by obesity-related gastroesophageal reflux, while squamous cell carcinoma is more linked to heavy alcohol and tobacco exposure [22], but our study implies that MASLD with increased alcohol exposure is clearly associated with the incidence of esophageal adenocarcinoma. Likewise, intestinal-type gastric cancers are associated with environmental and lifestyle factors such as smoking and alcohol [23], whereas diffuse-type gastric cancers more often reflect genetic predisposition or occur even in the absence of severe atrophic gastritis [24]. Our results reinforce these patterns: metabolic dysfunction predominantly heightens the risk of adenocarcinoma-type cancers, and alcohol acts as a co-carcinogen that further promotes those subtype-specific risks, which is in line with the results from esophageal adenocarcinoma. Importantly, heavy ethanol intake remains a major etiological factor for squamous carcinoma of the esophagus [22], but our results suggest that in MASLD populations, the excess UGI cancer burden seems to manifest chiefly in adenocarcinomas (esophageal and gastric intestinal) rather than in squamous or diffuse histologies.

However, statistical power varied considerably across cancer subtypes. Our primary findings for esophageal adenocarcinoma (643 total events) and intestinal gastric cancer (438 events) had adequate statistical power, with at least 200 total events and at least 10 events per SLD subgroup, yielding relatively narrow confidence intervals that support robust conclusions. In contrast, analyses of rarer subtypes, including squamous cell esophageal cancer (235 events, with only 9 in MASLD1) and other esophageal subtypes (41 events total, with 3 to 10 per subgroup), were underpowered, resulting in extremely wide confidence intervals. These latter findings should be considered exploratory and hypothesis-generating rather than definitive, requiring validation in larger, independent cohorts.

Multiple mechanisms likely underlie the heightened risk of upper GI adenocarcinomas in patients with MASLD and alcohol exposure. In MASLD, chronic insulin resistance, hyperinsulinemia, and elevated IGF-1 signaling promote epithelial proliferation and inhibit apoptosis, contributing to carcinogenesis in both the esophagus and stomach [25]. These pathways are implicated in the development of Barrett’s esophagus and esophageal adenocarcinoma, as well as gastric intestinal metaplasia and neoplasia. Dysregulation of adipokines—reduced adiponectin and increased TNF-α or IL-6—also fosters inflammation and angiogenesis, particularly in esophageal and gastric mucosa [26]. Additionally, oxidative stress and lipid peroxidation may induce DNA damage at distant organs including UGI tract [26]. On the other hand, alcohol exerts site-specific carcinogenic effects via direct mucosal contact [27], even in the mild alcohol exposure [28]. Acetaldehyde, its main metabolite, damages DNA and disrupts repair processes in esophageal and gastric tissues [12]. Furthermore, alcohol promotes barrier dysfunction and mutagenesis in gastric mucosa [29]. Thus, when combined with the pro-inflammatory, hyperinsulinemic state of MASLD, alcohol may act synergistically to promote adenocarcinogenesis, explaining the particularly elevated risks of esophageal and intestinal-type gastric cancers especially in MetALD and ALD populations.

Our study suggests notable clinical and public health implications. With global increases in obesity and alcohol use [30], more individuals are now classified as MASLD or MetALD, potentially elevating UGI cancer incidence. Our data suggest that not all fatty liver patients share equal risk—those with even mild to moderate alcohol consumption face significantly higher risk of esophageal and gastric adenocarcinomas than alcohol-abstinent MASLD patients. This distinction supports more tailored surveillance strategies. For instance, MASLD patients with alcohol exposure may benefit from early endoscopic evaluation, especially if reflux symptoms are present. Preventively, weight loss, metabolic control, and alcohol reduction may mitigate cancer risk [31]. Even low levels of alcohol intake have been linked to increased UGI cancer risk [32]. Public health strategies targeting obesity and alcohol misuse may offer additional cancer prevention benefits in high-risk populations.

Despite robust findings, several limitations merit consideration. First, MASLD and MetALD classifications relied on FLI and self-reported alcohol use, which may lead to misclassification—some MASLD individuals could underreport drinking, diluting risk estimates. Frequency-based alcohol categorization also lacks dose specificity. To address concerns about potential alcohol exposure misclassification, we performed sensitivity analyses additionally adjusting for serum GGT levels, an objective biomarker reflecting both alcohol metabolism and hepatic injury. Notably, the GGT-adjusted models (Table S4) showed even stronger associations between SLD subtypes and upper GI cancers compared to our primary analyses, particularly for esophageal adenocarcinoma and intestinal-type gastric cancer. This pattern suggests that self-reported alcohol consumption in our study may have actually underestimated true exposure levels, and our primary findings likely represent conservative estimates of the true associations. Nevertheless, the lack of quantitative alcohol intake data (grams per day) and information on drinking duration remains a limitation, as dose–response relationships could not be fully characterized. Despite these constraints, the consistency of associations across both self-report-based and biomarker- adjusted models strengthens confidence in our conclusions.

Second, our analysis lacks information on several important potential confounders, including Helicobacter pylori infection, gastroesophageal reflux disease, Barrett’s esophagus, and dietary patterns. While the UK Biobank contains some limited data on these variables for subsets of participants, they were not systematically collected at scale across the full cohort, precluding their inclusion in our analysis. H. pylori is a well-established risk factor for gastric cancer and associated with reduced risk of esophageal adenocarcinomas [33]. The absence of information on *H. pylori* from our dataset represents a significant limitation for interpreting esophageal and gastric cancer findings. The direction and magnitude of potential bias from these unmeasured factors are difficult to quantify without additional data. Future studies incorporating H. pylori serology, endoscopic assessments for GERD, Barrett’s esophagus, and validated dietary questionnaires are needed to more comprehensively evaluate these relationships.

Furthermore, exposure variables were assessed only at baseline without follow-up monitoring, so changes in alcohol consumption or metabolic status during the 13-year observation period were not captured, potentially leading to additional misclassification. Such temporal changes could theoretically inflate risk estimates if participants improved their exposures or dilute estimates if exposures worsened. However, regression dilution bias from non-differential misclassification typically attenuates associations toward the null, suggesting that our findings may underestimate rather than overestimate true risks.

Finally, as MASLD/MetALD definitions evolve, caution is needed when comparing with older NAFLD-based studies. Nonetheless, the consistent trend across cohorts that SLD combined with alcohol exposure confers the highest cancer risk reinforces the validity of our findings.

## 5. Conclusions

This large-scale UK Biobank cohort study demonstrates that UGI cancer risk in MASLD patients is significantly modified by alcohol consumption patterns. The refined SLD classification framework proves clinically valuable for risk stratification, identifying patients with combined metabolic dysfunction and alcohol exposure as having the highest risks for esophageal adenocarcinoma and intestinal-type gastric cancer. These findings support implementing tailored surveillance strategies and comprehensive interventions targeting both metabolic health and alcohol consumption to mitigate UGI cancer risk in high-risk populations.

## Figures and Tables

**Table 1:** Baseline Characteristics and Metabolic Profiles of Study Participants According to Steatotic Liver Disease Classification.

		Non-SLD	MASLD1	MASLD2	MetALD	ALD	*p* Value
		(N = 293,032)	(N = 13,996)	(N = 79,472)	(N = 35,905)	(N = 33,548)	
Age		55.82 (8.22)	57.18 (8.10)	56.51 (8.01)	56.88 (7.71)	58.10 (7.39)	<0.001
Sex	Male	107,355 (36.6)	6520 (46.6)	45,107 (56.8)	27,048 (75.3)	26,874 (80.1)	<0.001
	Female	185,677 (63.4)	7476 (53.4)	34,365 (43.2)	8857 (24.7)	6674 (19.9)	
Smoking status	Never	171,299 (58.8)	8419 (60.6)	42,175 (53.3)	16,583 (46.4)	11,691 (35.0)	<0.001
	Previous	90,585 (31.1)	3909 (28.2)	28,040 (35.4)	15,533 (43.4)	17,011 (50.9)	
	Current	29,458 (10.1)	1558 (11.2)	8890 (11.2)	3649 (10.2)	4734 (14.2)	
Weekly alcohol use	Never	22,749 (7.8)	13,996 (100.0)	0 (0.0)	0 (0.0)	0 (0.0)	<0.001
	<2	140,928 (48.3)	0 (0.0)	79,472 (100.0)	0 (0.0)	0 (0.0)	
	3–4	69,506 (23.8)	0 (0.0)	0 (0.0)	35,905 (100.0)	0 (0.0)	
	Almost daily	58,857 (20.2)	0 (0.0)	0 (0.0)	0 (0.0)	33,548 (100.0)	
Hypertension	No	258,708 (88.3)	11,504 (82.2)	66,082 (83.2)	30,258 (84.3)	27,822 (82.9)	<0.001
	Yes	34,324 (11.7)	2492 (17.8)	13,390 (16.8)	5647 (15.7)	5726 (17.1)	
Diabetes (all types)	No	283,144 (96.6)	11,025 (78.8)	67,812 (85.3)	32,171 (89.6)	30,183 (90.0)	<0.001
	Yes	9888 (3.4)	2971 (21.2)	11,660 (14.7)	3734 (10.4)	3365 (10.0)	
Dyslipidemia	No	267,785 (91.4)	11,788 (84.2)	68,662 (86.4)	31,116 (86.7)	28,750 (85.7)	<0.001
	Yes	25,247 (8.6)	2208 (15.8)	10,810 (13.6)	4789 (13.3)	4798 (14.3)	
BMI		25.19 (3.26)	32.77 (5.14)	32.16 (4.71)	30.66 (3.82)	30.06 (3.67)	<0.001
WC		83.58 (9.92)	103.80 (11.08)	103.00 (10.44)	101.82 (9.15)	101.82 (9.12)	<0.001
GGT		26.21 (20.62)	48.45 (51.10)	48.30 (46.93)	57.26 (54.44)	72.85 (79.11)	<0.001
Glucose		89.60 (16.92)	101.12 (37.94)	96.62 (30.18)	94.43 (24.37)	95.32 (23.81)	<0.001
Triglyceride		118.85 (55.44)	210.63 (108.10)	212.17 (105.74)	213.19 (105.62)	212.68 (108.91)	<0.001
HDL		60.53 (14.64)	45.71 (10.87)	46.70 (10.65)	49.34 (11.10)	52.48 (12.51)	<0.001

Variables are expressed as number (percentage). SLD, steatotic liver disease; MASLD, metabolic dysfunction-associated steatotic liver disease; MetALD, MASLD with moderate alcohol consumption; ALD, alcohol-associated liver disease; BMI, body mass index; WC, waist circumference; GGT, gamma-glutamyl transferase; HDL, high-density lipoprotein cholesterol.

**Table 2:** Incidence of Upper Gastrointestinal Cancers and All-Cause Mortality According to Steatotic Liver Disease Classification.

		Non-SLD	MASLD1	MASLD2	MetALD	ALD
End-point status	Subtype	(N = 293,032, 3,527,433 person-year)	(N = 13,996, 164,360 person-year)	(N = 79,472, 944,540 person-year)	(N = 35,905, 425,466 person-year)	(N = 33,548, 389,545 person-year)
Esophageal cancer	Overall	426 (12.08)	39 (23.73)	221 (23.40)	106 (24.91)	127 (32.60)
	Squamous	159 (4.51)	9 (5.48)	20 (2.12)	17 (4.00)	30 (7.70)
	Adeno	251 (7.12)	27 (16.43)	191 (20.22)	83 (19.51)	91 (23.36)
	Others	16 (0.45)	3 (1.83)	10 (1.06)	6 (1.41)	6 (1.54)
Gastric cancer	Overall	327 (9.27)	37 (22.51)	142 (15.03)	62 (14.57)	74 (19.00)
	Intestinal	203 (5.75)	27 (16.43)	106 (11.22)	45 (10.58)	57 (14.63)
	Non-intestinal	124 (3.52)	10 (6.08)	36 (3.81)	17 (4.00)	17 (4.36)
Death	Overall	19,385 (549.55)	2013 (1224.75)	8096 (857.14)	3310 (777.97)	3989 (1024.01)

Variables are expressed as number (incidence per 100,000 person-year). SLD, steatotic liver disease; MASLD, metabolic dysfunction-associated steatotic liver disease; MetALD, MASLD with moderate alcohol use; ALD, alcohol-associated liver disease.

**Table 3:** Hazard Ratios for Esophageal and Gastric Cancers According to Steatotic Liver Disease Classification Using Cox Proportional Hazard Model.

		Non-SLD	MASLD1	MASLD2	MetALD	ALD
			HR [95% CI]	HR [95% CI]	HR [95% CI]	HR [95% CI]
		Crude				
Esophageal cancer	Overall	(Ref)	**1.99 [1.43–2.76]**	**1.97 [1.68–2.32]**	**2.11 [1.70–2.61]**	**2.75 [2.25–3.35]**
	Squamous	(Ref)	1.23 [0.63–2.41]	0.48 [0.30–0.76]	0.91 [0.55–1.50]	1.74 [1.18–2.57]
	Adeno	(Ref)	**2.34 [1.57–3.49]**	**2.90 [2.40–3.50]**	**2.81 [2.19–3.60]**	**3.35 [2.63–4.26]**
	Others	(Ref)	**3.98 [1.16–13.66]**	**2.31 [1.05–5.09]**	**3.08 [1.20–7.86]**	**3.36 [1.31–8.58]**
Gastric cancer	Overall	(Ref)	**2.45 [1.74–3.44]**	**1.65 [1.36–2.01]**	**1.61 [1.22–2.11]**	**2.08 [1.62–2.68]**
	Intestinal	(Ref)	**2.87 [1.92–4.29]**	**1.98 [1.57–2.51]**	**1.88 [1.36–2.59]**	**2.57 [1.92–3.45]**
	Non-intestinal	(Ref)	1.75 [0.92–3.33]	1.11 [0.76–1.61]	1.17 [0.70–1.94]	1.27 [0.76–2.10]
		Adjusted				
Esophageal cancer	Overall	(Ref)	1.00 [0.66–1.53]	**1.60 [1.30–1.96]**	**1.41 [1.07–1.86]**	**1.49 [1.14–1.93]**
	Squamous	(Ref)	0.51 [0.24–1.09]	0.76 [0.45–1.30]	0.84 [0.47–1.50]	1.22 [0.76–1.97]
	Adeno	(Ref)	1.52 [0.86–2.69]	**1.77 [1.40–2.24]**	**1.80 [1.27–2.55]**	**1.67 [1.20–2.32]**
	Others	(Ref)	3.37 [0.35–32.71]	1.40 [0.54–3.65]	1.19 [0.38–3.79]	6.97 [0.83–58.49]
Gastric cancer	Overall	(Ref)	1.50 [0.93–2.40]	1.08 [0.86–1.35]	1.39 [0.97–1.99]	**1.55 [1.10–2.19]**
	Intestinal	(Ref)	1.67 [0.94–2.97]	1.21 [0.92–1.60]	**1.74 [1.10–2.73]**	**1.77 [1.17–2.68]**
	Non-intestinal	(Ref)	1.16 [0.50–2.70]	0.80 [0.53–1.23]	0.95 [0.51–1.76]	1.11 [0.59–2.11]

Adjusted for age, sex, smoking status, hypertension, diabetes, dyslipidemia, and weekly alcohol use. MASLD, metabolic dysfunction-associated steatotic liver disease; MetALD, MASLD with moderate alcohol consumption; ALD, alcohol-associated liver disease; HR, hazard ratio; CI, confidence interval.

**Table 4:** Hazard Ratios for Esophageal and Gastric Cancers According to Steatotic Liver Disease Classification Using the Cox Proportional Hazard Model, Stratified By Age.

		Non-SLD	MASLD1	MASLD2	MetALD	ALD
			HR [95% CI]	HR [95% CI]	HR [95% CI]	HR [95% CI]
		<60 years old				
Esophageal cancer	Overall	(Ref)	0.97 [0.47–2.00]	**1.51 [1.08–2.11]**	**2.19 [1.28–3.75]**	**1.72 [1.10–2.67]**
	Squamous	(Ref)	0.39 [0.08–1.85]	1.14 [0.50–2.59]	0.95 [0.32–2.84]	1.38 [0.66–2.91]
	Adeno	(Ref)	1.30 [0.53–3.21]	**1.49 [1.01–2.18]**	**3.11 [1.54–6.25]**	**1.96 [1.09–3.51]**
	Others	(Ref)	**-**	**-**	**-**	**-**
Gastric cancer	Overall	(Ref)	1.90 [0.72–5.02]	**1.50 [1.02–2.21]**	1.58 [0.86–2.90]	**2.05 [1.04–4.07]**
	Intestinal	(Ref)	7.97 [0.96–66.37]	**1.75 [1.09–2.81]**	1.69 [0.79–3.58]	**2.88 [1.17–7.13]**
	Non-intestinal	(Ref)	0.87 [0.24–3.13]	1.05 [0.52–2.11]	1.38 [0.49–3.89]	1.21 [0.40–3.65]
		≥60 years old				
Esophageal cancer	Overall	(Ref)	1.00 [0.60–1.68]	**1.63 [1.25–2.12]**	**1.16 [0.83–1.62]**	1.36 [0.98–1.88]
	Squamous	(Ref)	0.55 [0.23–1.32]	0.56 [0.27–1.16]	0.79 [0.40–1.57]	1.12 [0.61–2.08]
	Adeno	(Ref)	1.65 [0.79–3.46]	**1.94 [1.43–2.62]**	1.43 [0.95–2.16]	**1.52 [1.02–2.26]**
	Others	(Ref)	2.18 [0.20–24.39]	1.02 [0.25–4.20]	0.84 [0.22–3.21]	3.23 [0.33–31.52]
Gastric cancer	Overall	(Ref)	1.39 [0.81–2.38]	0.89 [0.67–1.19]	1.31 [0.84–2.04]	1.40 [0.94–2.09]
	Intestinal	(Ref)	1.35 [0.73–2.50]	1.00 [0.71–1.41]	**1.80 [1.02–3.18]**	1.54 [0.97–2.45]
	Non-intestinal	(Ref)	1.44 [0.46–4.49]	0.68 [0.39–1.17]	0.77 [0.35–1.67]	1.06 [0.48–2.33]

Adjusted for age, sex, smoking status, hypertension, diabetes, dyslipidemia, and weekly alcohol use. MASLD, metabolic dysfunction-associated steatotic liver disease; MetALD, MASLD with moderate alcohol consumption; ALD, alcohol-associated liver disease; HR, hazard ratio; CI, confidence interval.

## Data Availability

The data used in this study were obtained from the UK Biobank under application number 435869. Access to the data is available to researchers upon approved application at https://www.ukbiobank.ac.uk.

## Supplementary Materials

The following supporting information can be downloaded at https://www.mdpi.com/article/10.3390/cancers17213416/s1, Supplementary Figure S1. Directed Acyclic Graph to Explore Confounders between Steatotic Liver Disease and Gastrointestinal Cancers; Supplementary Figure S2. Cumulative Incidence of Esophageal and Gastric Cancers Subtypes by MASLD Subtype Over 13 Years of Follow-up; Supplementary Table S1. Hazard Ratios for Esophageal and Gastric Cancers According to Steatotic Liver Disease Classification using Fine–Gray Competing Risk Analysis; Supplementary Table S2. Absolute Risk and Risk Differences for Esophageal Adenocarcinoma and Intestinal-Type Gastric Cancer by SLD Classification; Supplementary Table S3. Variance Inflation Factor Analysis for Multicollinearity Detection in Multivariate Cox Regression Model; Supplementary Table S4. Hazard Ratios for Esophageal and Gastric Cancers According to Steatotic Liver Disease Classification, Additionally Adjusted for Serum Gamma-Glutamyl Transferase Levels.

## Abbreviations

ALD (alcoholic liver disease), BMI (body mass index), CI (confidence interval), DAG (directed acyclic graph), FLI (fatty liver index), GGT (gamma-glutamyl transferase), GI (gastrointestinal), HDL (high-density lipoprotein), HR (hazard ratio), ICD-O-3 (International Classification of Diseases for Oncology, 3rd Edition), MASLD (metabolic dysfunction-associated steatotic liver disease), MASLD1 (MASLD with no alcohol use), MASLD2 (MASLD with minimal alcohol use), MetALD (Metabolic and alcohol-associated liver disease), sHR (subdistribution hazard ratio), SLD (steatotic liver disease), UGI (upper gastrointestinal), UK (United Kingdom), WC (waist circumference).
