# Supplementary material for: Risk of Esophageal and Gastric Cancer by Histologic Subtype in Steatotic Liver Disease: A UK Biobank Study
Source: Cancers (Basel). 2025 Oct 24;17(21):3416. doi: 10.3390/cancers17213416 (PMC12609825; doi:10.3390/cancers17213416)
Supplement: Supplementary file 1 [file cancers-17-03416-s001.zip › Table S1.pdf]

Supplementary Table 1. Hazard Ratios for Esophageal and Stomach Cancers According to Steatotic Liver Disease Classification using Fine-Gray Competing Risk Analysis

		Non-SLD	MASLD_1		MASLD_2		MetALD		ALD	
			sHR	95% CI	sHR	95% CI	sHR	95% CI	sHR	95% CI
		Crude								
Esophageal cancer	Overall	(Ref)	1.92	[1.39–2.67]	1.92	[1.63–2.26]	2.03	[1.64–2.52]	2.61	[2.14–3.18]
	Squamous	(Ref)	1.19	[0.61–2.32]	0.46	[0.29–0.74]	0.87	[0.53–1.44]	1.65	[1.11–2.43]
	Adeno	(Ref)	2.26	[1.52–3.37]	2.82	[2.33–3.40]	2.71	[2.11–3.47]	3.18	[2.50–4.04]
	Others	(Ref)	3.93	[1.14–13.48]	2.30	[1.04–5.07]	3.06	[1.20–7.80]	3.27	[1.28–8.36]
Stomach cancer	Overall	(Ref)	2.38	[1.69–3.34]	1.60	[1.32–1.95]	1.55	[1.18–2.03]	1.98	[1.54–2.55]
	Intestinal	(Ref)	2.79	[1.87–4.17]	1.92	[1.52–2.43]	1.80	[1.31–2.49]	2.45	[1.83–3.28]
	Non-intestinal	(Ref)	1.70	[0.89–3.23]	1.08	[0.74–1.56]	1.12	[0.68–1.87]	1.20	[0.72–2.00]
		Adjusted								
Esophageal cancer	Overall	(Ref)	1.38	[0.95-2.00]	1.39	[1.16-1.67]	1.36	[1.09-1.69]	1.57	[1.22-2.02]
	Squamous	(Ref)	1.17	[0.55-2.48]	0.47	[0.29-0.77]	0.87	[0.52-1.47]	1.38	[0.86-2.22]
	Adeno	(Ref)	1.43	[0.91-2.25]	1.81	[1.46-2.23]	1.57	[1.21-2.03]	1.72	[1.26-2.34]
	Others	(Ref)	1.87	[0.47-7.48]	1.39	[0.59-3.31]	2.11	[0.77-5.75]	2.60	[0.75-8.98]
Stomach cancer	Overall	(Ref)	1.36	[0.93-2.00]	1.10	[0.90-1.36]	1.17	[0.88-1.55]	1.61	[1.17-2.21]
	Intestinal	(Ref)	1.51	[0.95-2.38]	1.28	[0.99-1.64]	1.31	[0.93-1.83]	1.93	[1.31-2.85]
	Non-intestinal	(Ref)	1.08	[0.53-2.22]	0.79	[0.54-1.17]	0.93	[0.55-1.58]	1.06	[0.58-1.93]

Adjusted for age, sex, smoking status, hypertension, diabetes, dyslipidemia, weekly alcohol use
