# Supplementary material for: Risk of Esophageal and Gastric Cancer by Histologic Subtype in Steatotic Liver Disease: A UK Biobank Study
Source: Cancers (Basel). 2025 Oct 24;17(21):3416. doi: 10.3390/cancers17213416 (PMC12609825; doi:10.3390/cancers17213416)
Supplement: Supplementary file 1 [file cancers-17-03416-s001.zip › Table S2.pdf]

Supplementary Table 2. Absolute Risk and Risk Differences for Esophageal Adenocarcinoma and Intestinal-Type Gastric Cancer by SLD Classification

Cancer Type		Non-SLD	MASLD1	MASLD2	MetALD	ALD
Esophageal Adenocarcinoma	AR	7.12	16.43	20.22	19.51	23.36
	ARD	(Ref)	+ 9.31	+ 13.10	+ 12.39	+ 16.24
Intestinal Type Gastric Cancer	AR	5.75	16.43	11.22	10.58	14.63
	ARD	(Ref)	+ 10.68	+ 5.47	+ 4.83	+ 8.88

AR, absolute risk; ARD, absolute risk difference. Values represent cases per 100,000 person-years. SLD, steatotic liver disease; MASLD, metabolic dysfunction-associated steatotic liver disease; MetALD, MASLD with moderate alcohol consumption; ALD, alcohol-associated liver disease.
