# Supplementary material for: Risk of Esophageal and Gastric Cancer by Histologic Subtype in Steatotic Liver Disease: A UK Biobank Study
Source: Cancers (Basel). 2025 Oct 24;17(21):3416. doi: 10.3390/cancers17213416 (PMC12609825; doi:10.3390/cancers17213416)
Supplement: Supplementary file 1 [file cancers-17-03416-s001.zip › Table S3.pdf]

Supplementary Table 3. Variance Inflation Factor Analysis for Multicollinearity Detection in Multivariate Cox Regression Model

	GVIF	Df	GVIF^(1/(2*Df))
ALD classification	9.599	4	1.327
Age	1.046	1	1.023
Sex	1.101	1	1.049
Smoking status	1.082	2	1.020
Hypertension	1.040	1	1.020
Diabetes	1.070	1	1.034
Dyslipidemia	1.041	1	1.021
Weekly alcohol use	8.940	3	1.441

*ALD, alcohol-associated liver disease.
