# Supplementary material for: Risk of Esophageal and Gastric Cancer by Histologic Subtype in Steatotic Liver Disease: A UK Biobank Study
Source: Cancers (Basel). 2025 Oct 24;17(21):3416. doi: 10.3390/cancers17213416 (PMC12609825; doi:10.3390/cancers17213416)
Supplement: Supplementary file 1 [file cancers-17-03416-s001.zip › Table S4.pdf]

Supplementary Table 4. Hazard Ratios for Esophageal and Gastric Cancers According to Steatotic Liver Disease Classification, Additionally Adjusted for Serum Gamma-Glutamyl Transferase Levels

		**Non-SLD**	**MASLD1**	**MASLD2**	**MetALD**	**ALD**
			HR [95% CI]	HR [95% CI]	HR [95% CI]	HR [95% CI]
Esophageal cancer	Overall	(Ref)	**1.53 [1.09–2.13]**	**1.42 [1.19–1.69]**	**1.26 [1.01–1.58]**	**1.32 [1.06–1.64]**
	Squamous	(Ref)	0.93 [0.47–1.85]	0.39 [0.24–0.63]	0.74 [0.44–1.24]	1.12 [0.73–1.74]
	Adeno	(Ref)	**1.85 [1.23–2.78]**	**2.03 [1.66–2.50]**	**1.55 [1.19–2.02]**	**1.48 [1.14–1.93]**
	Others	(Ref)	2.63 [0.73–9.47]	1.51 [0.64–3.54]	1.74 [0.64–4.75]	1.57 [0.56–4.42]
Gastric cancer	Overall	(Ref)	**2.02 [1.42–2.86]**	**1.29 [1.05–1.59]**	1.09 [0.82–1.45]	1.19 [0.90–1.58]
	Intestinal	(Ref)	**2.35 [1.56–3.54]**	**1.51 [1.18–1.94]**	1.20 [0.85–1.68]	1.35 [0.98–1.87]
	Non-intestinal	(Ref)	1.46 [0.75–2.84]	0.92 [0.61–1.37]	0.92 [0.54–1.58]	0.91 [0.52–1.58]

*Adjusted for age, sex, smoking status, hypertension, diabetes, dyslipidemia, serum GGT level.
MASLD, metabolic dysfunction-associated steatotic liver disease; MetALD, MASLD with moderate alcohol consumption; ALD, alcohol-associated liver disease; HR, hazard ratio; CI, confidence interval.
