# Supplementary figures and images for: Risk of Esophageal and Gastric Cancer by Histologic Subtype in Steatotic Liver Disease: A UK Biobank Study
Source: Cancers (Basel). 2025 Oct 24;17(21):3416. doi: 10.3390/cancers17213416 (PMC12609825; doi:10.3390/cancers17213416)

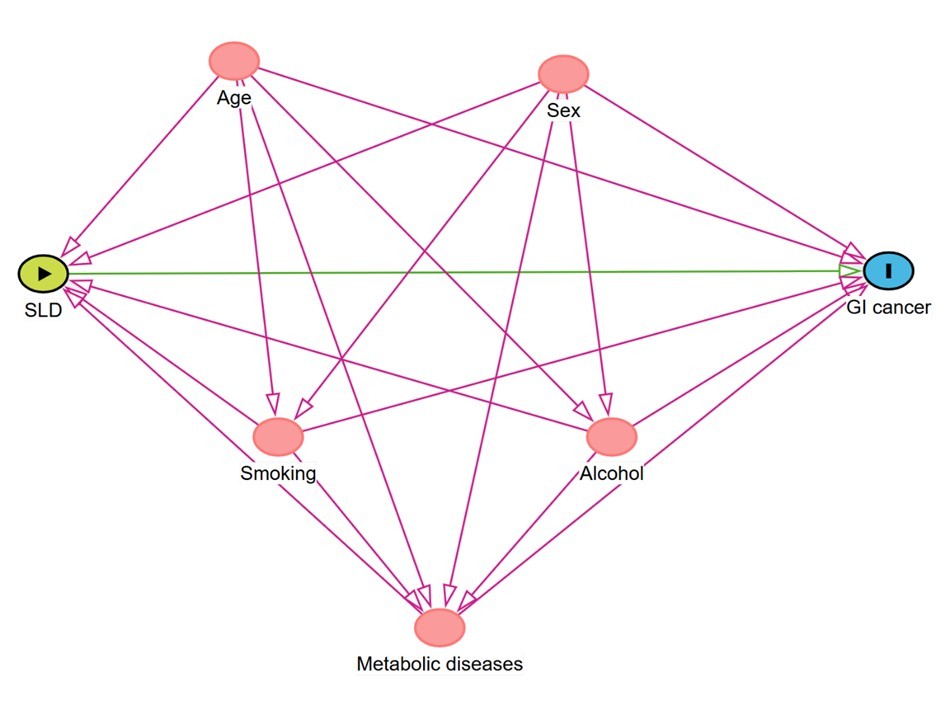

Supplement: Supplementary file 1 [file cancers-17-03416-s001.zip › Supplementary figure S1.JPG]

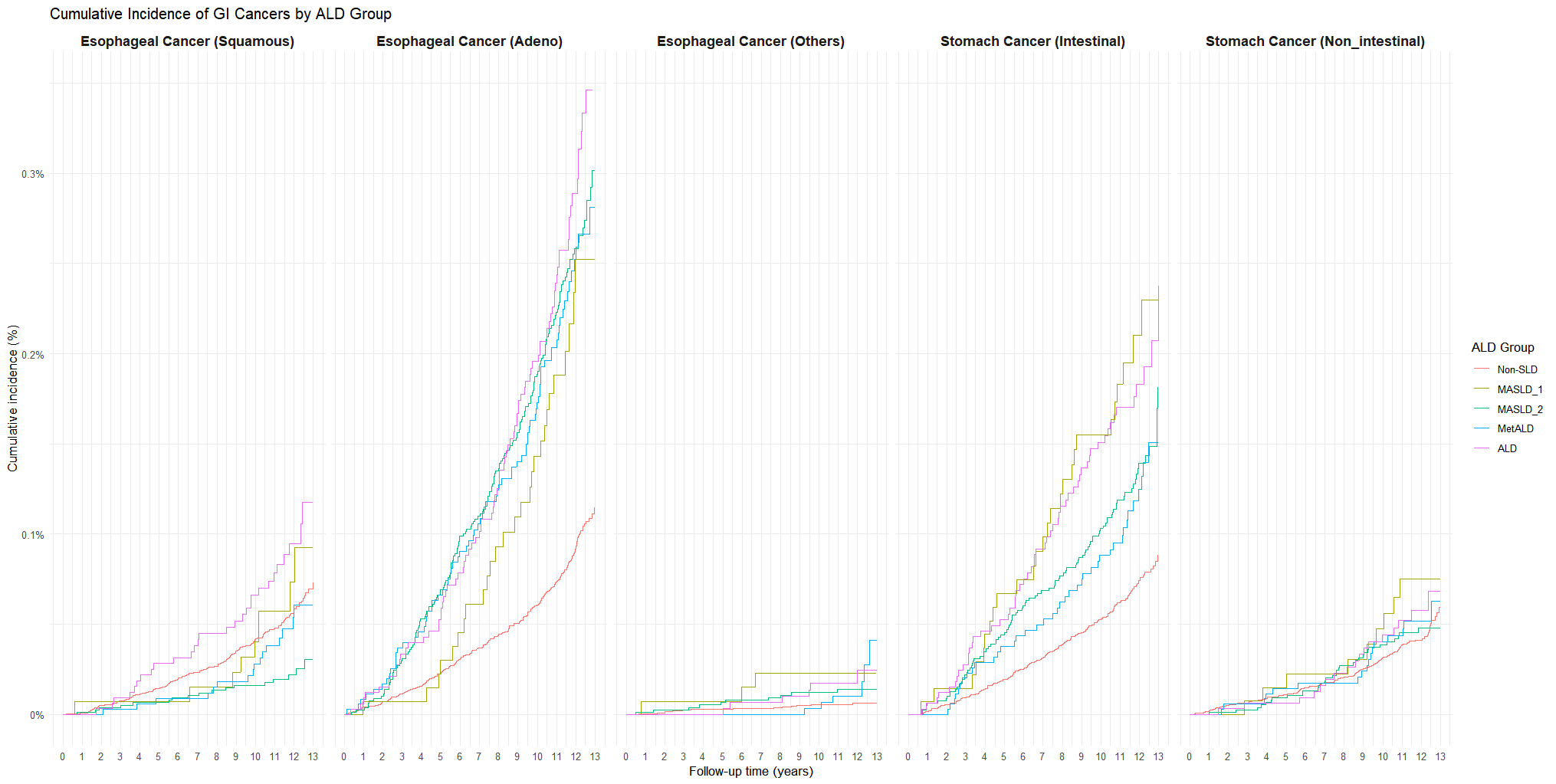

Supplement: Supplementary file 1 [file cancers-17-03416-s001.zip › Supplementary figure S2.png]
